# Reduced Amygdala and Ventral Striatal Activity to Happy Faces in PTSD Is Associated with Emotional Numbing

**DOI:** 10.1371/journal.pone.0103653

**Published:** 2014-09-03

**Authors:** Kim L. Felmingham, Erin M. Falconer, Leanne Williams, Andrew H. Kemp, Adrian Allen, Anthony Peduto, Richard A. Bryant

**Affiliations:** 1 School of Psychology, University of New South Wales, Sydney, Australia; 2 Brain Dynamics Centre, Westmead Millennium Institute, Westmead Hospital, Sydney, Australia; 3 School of Psychology, University of Tasmania, Hobart, Australia; 4 Western Clinical School, University of Sydney, Sydney, Australia; 5 School of Psychology, University of Sydney, Sydney, Australia; 6 MRI Unit, Department of Radiology, Westmead Hospital, Sydney, Australia; Yale University, United States of America

## Abstract

There has been a growing recognition of the importance of reward processing in PTSD, yet little is known of the underlying neural networks. This study tested the predictions that (1) individuals with PTSD would display reduced responses to happy facial expressions in ventral striatal reward networks, and (2) that this reduction would be associated with emotional numbing symptoms. 23 treatment-seeking patients with Posttraumatic Stress Disorder were recruited from the treatment clinic at the Centre for Traumatic Stress Studies, Westmead Hospital, and 20 trauma-exposed controls were recruited from a community sample. We examined functional magnetic resonance imaging responses during the presentation of happy and neutral facial expressions in a passive viewing task. PTSD participants rated happy facial expression as less intense than trauma-exposed controls. Relative to controls, PTSD participants revealed lower activation to happy (-neutral) faces in ventral striatum and and a trend for reduced activation in left amygdala. A significant negative correlation was found between emotional numbing symptoms in PTSD and right ventral striatal regions after controlling for depression, anxiety and PTSD severity. This study provides initial evidence that individuals with PTSD have lower reactivity to happy facial expressions, and that lower activation in ventral striatal-limbic reward networks may be associated with symptoms of emotional numbing.

## Introduction

PTSD is a complex psychiatric condition with features that extend beyond conditioned fear responses to include a wider range of emotional difficulties, such as emotional numbing [1]. Emotional numbing focuses on diminished *positive* affect, including an absence of feelings of love and happiness, and a lack of response to positive environmental events [2], [3]. Little is known about the neural networks associated with processing positive stimuli in PTSD. Recently, there has been a growing recognition of the importance of reward processing and in understanding its neural basis in PTSD. Emotional numbing has been shown to be the symptom that is most characteristic of chronic PTSD [4], and is strongly associated with functional and interpersonal impairment [5], [6].

Two behavioral studies have reported deficient reward function in PTSD. Vietnam veterans with PTSD have been found to expend less effort in viewing beautiful female faces [7], and to have lower expectancy and satisfaction with rewards in a gambling task [8]. Two fMRI studies have examined functioning of reward networks in PTSD using monetary reward tasks [9], [10]. Participants with PTSD showed lower activation in nucleus accumbens and medial prefrontal cortex (mPFC) [10] and in dorsal and ventral striatal regions (caudate and putamen) [9] compared to non-trauma exposed controls in response to gains. This pattern was interpreted to reflect deficiencies in reward function. However, it is unclear in these studies whether the lower activation in ventral striatal regions is due to PTSD specifically, or trauma-exposure, as both studies did not include a trauma-exposed control group.

Interestingly, anhedonia in patients with major depression has also been associated with lower responses in left nucleus accumbens and bilateral caudate in response to gains in a monetary incentive task [11]. Anhedonia, defined as a lack of reactivity to pleasurable stimuli, has considerable conceptual overlap to emotional numbing in PTSD. This raises the possibility that the lower ventral striatal activity seen in PTSD may at least partially be explained by the high rates of comorbid depression observed with this disorder.

Previous imaging studies in healthy controls have examined ventral striatal reactivity to gains in monetary incentive tasks [9]–[11], which may recruit distinctive networks from those involved in the perception of positive signals [12]. Perception of positive signals/hedonic tone appears centered on networks encompassing the nucleus accumbens [12], [13], whereas networks involved in processing stimulus-reward association are associated with wider orbitofrontal-dorsal and ventral striatal networks [13]. Therefore, it is important to examine reward function in ventral striatal networks in PTSD in terms of reactivity to positive stimuli. Only one pilot study has examined neural reactivity to positive affective signals (animated film) in PTSD. Greater activation was found in right precentral and superior frontal gyrus, and lower activation in parahippocampal and superior temporal gyrus were found in the PTSD group [14]. However, this study employed small sample sizes (8 males) and did not specifically examine emotional numbing.

In the healthy brain, happy faces engage a neural network involving the ventral striatum (encompassing putamen and nucleus accumbens) [15], amygdala [16], [17], [18], orbitofrontal cortex [19] and anterior cingulate [16]. To date, the neural correlates of perception of innate positive signals, such as happy facial expressions, have not been examined in PTSD. Examining reactivity to facial expressions in PTSD is of particular importance, as research suggests that deficiencies in response to facial displays of affect contribute significantly to functional and social impairment [20]. Indeed, emotional numbing, which involves blunted positive affect and reactivity, has been found as the major contributor to functional impairment in PTSD [5], [6].

Accordingly, the present study examined the neural networks underlying the processing of happy facial expressions in PTSD. Secondly, we examined the extent to which impairments in reactivity to positive emotional signals were associated with emotional numbing in PTSD. It was predicted there would be a lower activation in networks involved in the processing of happy faces (ventral striatum encompassing nucleus accumbens, and amygdala) in PTSD compared to trauma-exposed controls. Further, we expected lower activation in these regions to be associated with more severe symptoms of emotional numbing in PTSD.

## Methods and Materials

### Participants

Twenty-three individuals who developed PTSD as a result of physical assault (n = 15) or motor vehicle accidents (n = 8) were recruited for the study from the Traumatic Stress Clinic, Westmead Hospital. There were 13 female and 10 male participants with an average duration since trauma of 60.8 months (SD = 74.3). Twenty trauma-exposed controls (Trauma Controls: those who had experienced a criterion A trauma (defined by DSM-IV as being confronted with an experience that threatened physical integrity) but did not develop PTSD symptoms that reached diagnostic PTSD (or sub-clinical status) were recruited from community settings (in collaboration with the Brain Resource International Database) [21]. Ten Trauma Controls had experienced motor vehicle accidents, and 10 had experienced interpersonal assault. There was an average duration post-trauma of 110 months (SD = 139) for Trauma Controls. Participants were administered the Composite International Diagnostic Interview (CIDI) [22] to diagnose those with PTSD and identify those who were trauma-exposed controls. Trauma Controls reported symptoms that did not meet criteria for the Re-experiencing cluster (cluster B) of symptoms, or more than one PTSD symptom cluster for Avoidance or Hyperarousal. To provide an estimate of the severity and frequency of PTSD symptoms (including emotional numbing), participants with PTSD were also administered the Clinician Administered PTSD Scale (CAPS) [23]. Fifteen participants with PTSD had comorbid major depression (8 were medicated with antidepressants), 1 had comorbid panic disorder, and 1 comorbid OCD. All participants were excluded if there was any current substance abuse or dependence (within six months of testing), history of brain injury or neurological condition, psychosis or significant medical condition. To provide an estimate of current mood, all participants were administered the Depression Anxiety Stress Scales [24]. All participants provided written informed consent according to the Declaration of Helsinki. This study was approved by the Western Sydney Area Health Service Human Research Ethics Board.

### Face Emotion Perception Task

The facial emotion perception task was previously established to activate networks in ventral striatum, anterior cingulate cortex and amygdala [25]. Participants viewed 240 grey-scale face stimuli selected from a standardized picture set [26]. The set consisted of four female and four male individuals depicting happy and neutral facial expressions. Face stimuli were presented in a pseudorandom sequence of 30 blocks (comprising 8 happy or 8 neutral faces each). Each stimulus was presented for 500 ms and was followed by a 767.5 ms blank screen interstimulus interval. Emotion identification accuracy and ratings of the intensity of facial emotion were assessed immediately post-scanning. Intensity ratings employed 9-point Likert scales (0 = *not at all intense*, 9 = *very intense*).

### fMRI Data Acquisition

MRI scans were performed on a 1.5 T Siemens Vision Plus Scanner using an echo echoplanar protocol. A total of 90 functional T*2-weighted volumes (3 stimuli per block) were acquired for each condition, comprising 15 non-contiguous slices parallel to the intercommissural (AC-PC) line, with a 6.6 mm thickness and TR = 3.3 sec, TE = 40 ms, Flip angle = 90°; with FOV 24×24 cm^2^, matrix size 128×128.

### Functional MRI Data Reduction and Analysis

Pre-processing and statistical analysis of fMRI data was conducted using Statistical Parametric Mapping (SPM-2, Wellcome Department of Neurology, London, UK). Functional scans were realigned, unwarped, spatially normalized and smoothed in order to remove movement artefact and to place data into a common anatomical frame. Images were normalized into standardized MNI space and smoothed using a Gaussian kernel (FWHM: 8 mm).

An HRF-convolved boxcar model with temporal derivative was created to correspond to the experimental model, and a high pass filter was applied to remove low frequency fluctuations in the BOLD signal. Individual contrast images (fear versus neutral) were brought to the second level and examined using t-tests.

For the group voxelwise analysis, random effects independent samples t-tests comparing the PTSD and Trauma Control group to happy (-neutral) faces were conducted. To test our a priori hypotheses, analyses were undertaken using a search region of interest (ROI) approach which included regions of the amygdala and ventral striatum (caudate and putamen, including nucleus accumbens). Search regions were defined by the Automated Anatomical Labelling (AAL) Masks [27] and selected using the WFU Pickatlas (Version 1.02). We created an anatomical region of interest mask for the ventral striatum by combining caudate and putamen (including nucleus accumbens). Nucleus accumbens was identified at the inferior junction between the head of caudate and putamen, in accordance with previous studies and the Human Brain Atlas [28], [29]. Coordinates of peak activations within the nucleus accumbens were confirmed with reference to the Human Brain Atlas.

Previous studies of facial emotion processing have used an uncorrected *p* value of *p*<.05 for determining significant activations in small structures based on a priori hypotheses [17], or p<.005 when examining ventral striatum in monetary incentive tasks [11]. Given our a priori directional hypotheses, in line with previous literature [9], [17] we employed independent samples t-tests using the above ROIs, with an alpha value of p<.005, and a spatial extent threshold of 10 contiguous voxels. To examine the influence of comorbid depression, a sub-analysis (using the same analysis techniques as above) was conducted comparing PTSD_with depression (n = 15) and Trauma Controls, PTSD_no depression (n = 8) and Trauma Controls, and PTSD_with and PTSD_No depression groups using independent samples t-tests.

For the correlational analysis, intensity beta values were extracted from the most significant voxels for each individual in the ventral striatum ROI. Pearson Product moment correlations were conducted in SPM2 within the PTSD group between each individual's peak BOLD ventral striatal activity in response to happy faces and their emotional numbing score (derived from ratings on the CAPS in a procedure taken from Foa et al., (1995). To control for PTSD severity, depression and state anxiety, correlations were conducted with PTSD severity (total CAPS score), DASS depression score, and DASS anxiety score included as covariates. A probability estimate of *p*<.01 and the extent threshold >10 voxels per cluster was adopted.

## Results

### Demographic and Clinical Data

Demographic and clinical data are summarized in Table 1. The PTSD group were significantly older than the Trauma Controls, and there was a trend for Trauma Controls to have longer time since trauma, but there were no significant differences in gender distribution between groups. All analyses reported below were repeated taking age and time-post trauma as covariates; findings remained consistent and will not be reported. As expected, PTSD participants scored higher on the DASS depression and DASS anxiety scores.

**Table 1:** Summary of demographic and clinical characteristics of the PTSD (n = 23) and Trauma Exposed Control (n = 20) groups.

Variable	TC	PTSD	Test Statistic	p
Age	30.5(12)	38.5(11.5)	F = 5	.03*
Gender	11 M, 9 F	10 M, 13 F	χ^2^ = .57	.55
Time post-trauma (mths)	110 (139)	60.8 (74.3)	F = 3.8	.055
Trauma Type	10 MVA 10Assault	8 MVA 15Assault	χ^2^ = 1.09	.242
Comorbidity	Nil	15 MDD, 1 PD, 1 OCD	χ^2^ = 8.6	.004*
Medication	Nil	8 medicated 15unmedicated	χ^2^ = 8.6	.004*
DASS Dep	3.1 (2)	10.9 (5.2)	F = 34.5	.001*
DASS Anx	1.6(1.9)	13.1 (6.7)	F = 55	.001*

Note: Standard deviations in parentheses. TC = trauma-exposed controls, PTSD = Posttraumatic Stress Disorrder; M = male, F = female; mths = months; MVA = motor vehicle accident; MDD = major depressive disorder, PD = Panic Disorder, OCD = Obsessive Compulsive Disorder; DASS = Depression Anxiety and Stress Scale; dep = depression, anx = anxiety.

*p<.05,

**p<.001.

### Facial Expression Ratings Data

There were no significant differences between groups in accuracy for identification of happy (Trauma Control: 100%; PTSD: 99% correct) or neutral facial expressions (Trauma Control: 76%; PTSD: 78% correct). In terms of intensity of emotional expression, the PTSD group rated the happy faces as less intense than Trauma Controls (*F*_(1,26)_ = 4.2, *p*<.05).

### fMRI Data

**Figure 1:**
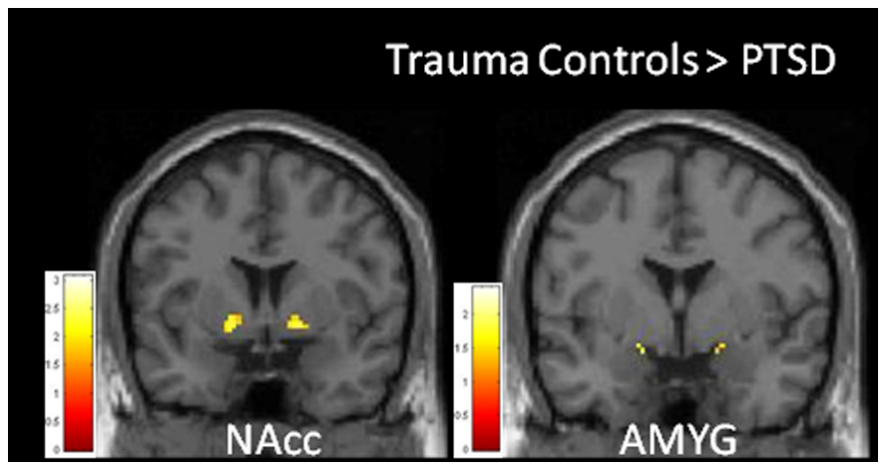
Reduced BOLD Activity in Ventral Striatum and Amygdala in PTSD (n = 23) compared to trauma-exposed controls (n = 20) in response to happy (- neutral) facial expressions.

**Figure 2:**
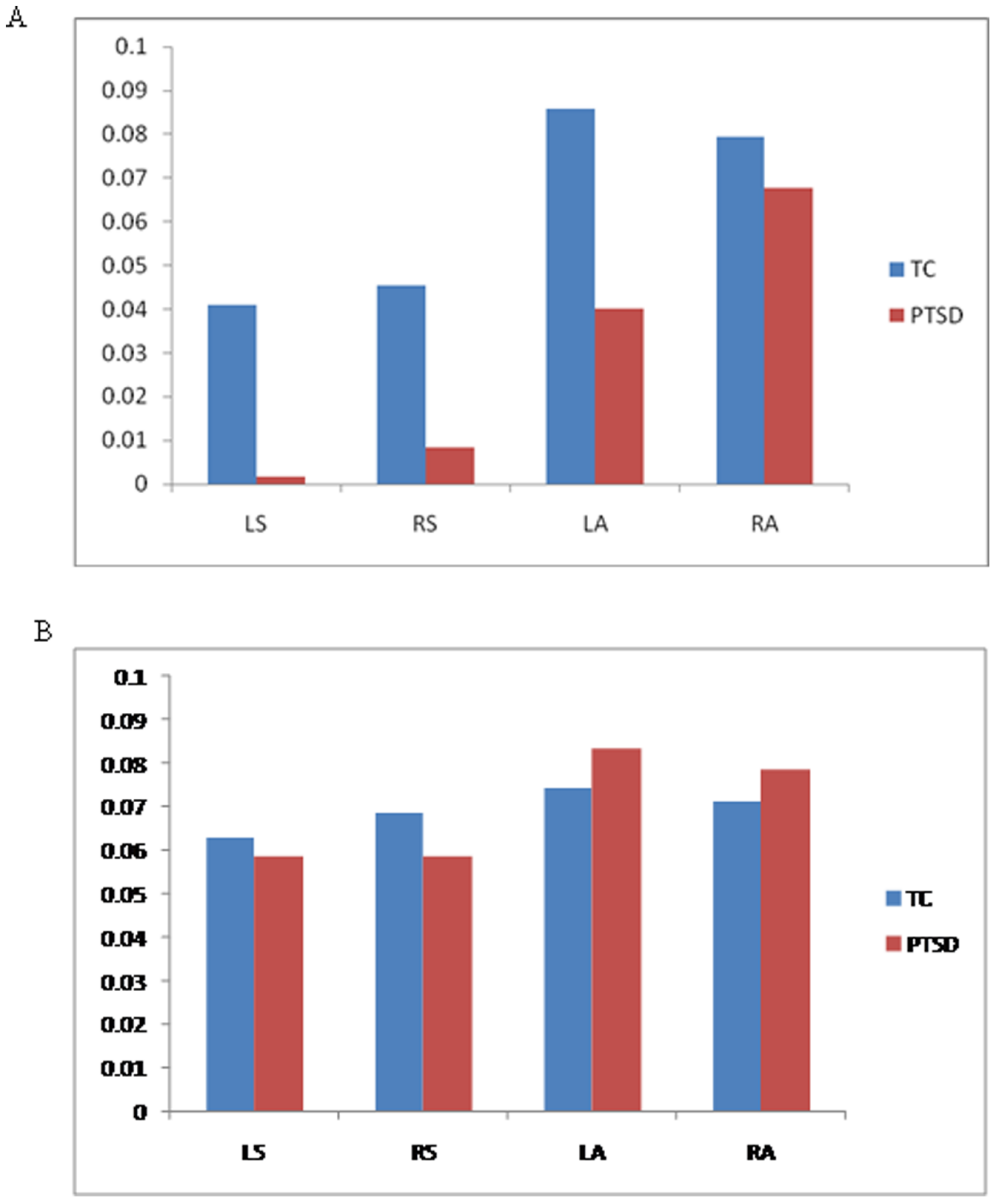
Percentage signal change in BOLD response to happy faces (Panel A) in Left Ventral Striatum (LS), Right ventral striatum (RS), Left Amygdala (LA), and Right Amygdala (RA), and percentage signal change in BOLD response to Neutral Faces (Panel B) in Left Ventral Striatum (LS), Right Ventral Striatum (RS), Left Amygdala (LA) and Right Amygdala (RA).

PTSD participants revealed lower activation in response to perception of happiness (versus neutral) left nucleus accumbens extending into putamen, and trends for lower activation in left amygdala compared to Trauma Controls (see Table 2 and Figure 1). There were no significantly greater activations in these regions of interest in the PTSD group. Examination of percentage of signal change to happy and neutral faces (see Figure 2) reveals that these group differences are most apparent in response to happy faces.

**Table 2:** Summary of between-group t-tests: Increased BOLD signal elicited by happy faces compared to neutral faces in the PTSD (n = 23) and Trauma-exposed controls (TC: n = 20; (p<.005 uncorrected).

Brain Region	Hem	MNI Coordinates			Voxels	t	p
		X	Y	Z			
***TC>PTSD***							
Amygdala	Left	−16	−2	−12	7	2.81	.05*
Ventral Striatum	Left	−14	0	−6	118	3.61	.000

Note: TC = trauma-exposed controls,

*trend level result.

#### Effect of Medication

To examine the impact of medication, fMRI data was reanalyzed comparing unmedicated PTSD patients (n = 15) with Trauma Controls, and comparing medicated PTSD patients (n = 8) with Trauma Controls (see Table 3). Findings reinforced our overall group findings. Unmedicated PTSD patients displayed lower activations in left amygdala and displayed trends for lower activation in right nucleus accumbens. There were no greater activations in these regions in the PTSD group. Further, a sub-analysis of fMRI data in medicated PTSD patients (n = 8) compared to Trauma Controls revealed trends for reduced activation in right amygdala and right ventral striatum.

**Table 3:** Increased BOLD signal elicited by happy faces compared to neutral faces in the PTSD-No Medication Group (n = 15) vs Trauma-exposed controls (TC: n = 20;), and PTSD-Medication Group (n = 8) vs Trauma-exposed controls (TC: n = 20).

Brain Region	Hem	MNI Coordinates			Voxels	t	p
***TC>PTSD_no medication***							
Amygdala	L	−16	−2	−12	18	2.81	.004
Ventral Striatum	R	18	6	−6	110	2.60	.009
***PTSD-no medication>TC***							
Amygdala	-	-	-	-	-	-	-
Ventral Striatum	-	-	-	-	-	-	-
***TC>Medicated PTSD***							
Amygdala	R	30	−4	−28	7	2.23	.017
Ventral Striatum	R	24	20	2	49	2.32	.014
***Medicated PTSD>TC***							
Amygdala	-	-	-	-	-	-	-
Ventral Striatum	-	-	-	-	-	-	-

Note: TC = trauma-exposed controls,

#### Effect of Depression

To examine the impact of depression, fMRI data was reanalyzed comparing PTSD with depression with Trauma Controls, and PTSD without depression to Trauma Controls. Findings are summarized in Table 4. Relative to Trauma Controls, the PTSD without depression group displayed significantly reduced activity in right amygdala and a trend for reduced activity in left ventral striatum. There were no greater activations in the PTSD group without depression over Trauma Controls. The PTSD group with depression revealed trends for reduced activation in right amygdala and significantly reduced activation in right ventral striatum. Comparing PTSD samples with and without depression revealed that PTSD patients with depression had lower activation in right caudate compared to PTSD without depression patients. PTSD patients without depression displayed no significant differences in neural activation compared to PTSD with depression patients.

**Table 4:** Increased BOLD signal elicited by happy faces compared to neutral faces in the PTSD-No Depression Group (n = 8) vs Trauma-exposed controls (TC: n = 20;), and PTSD-Depression Group (n = 15) vs Trauma-exposed controls (TC: n = 20).

Brain Region	Hem	MNI Coordinates			Voxels	t	p
***TC≥PTSD-no depression***							
Amygdala	R	22	−6	−12	18	2.49	.005
Ventral Striatum	L	14	4	−6	74	2.10	.009
***TC≥PTSD -depression***							
Amygdala	R	34	−2	−24	22	2.24	.008
Ventral Striatum	R	34	−16	0	120	2.93	.002
***Non-Depressed PTSD≥Depressed PTSD***							
Amygdala	R	32	−4	−20	11	2.31	.008
Ventral Striatum	R	10	14	12	843	6.19	.000
***Depressed PTSD≥Non-Depressed PTSD***							
Amygdala	-	-	-	-	-	-	-
Ventral Striatum	-	-	-	-	-	-	-

Note: TC = trauma-exposed controls,

#### Correlation Analyses

**Figure 3:**
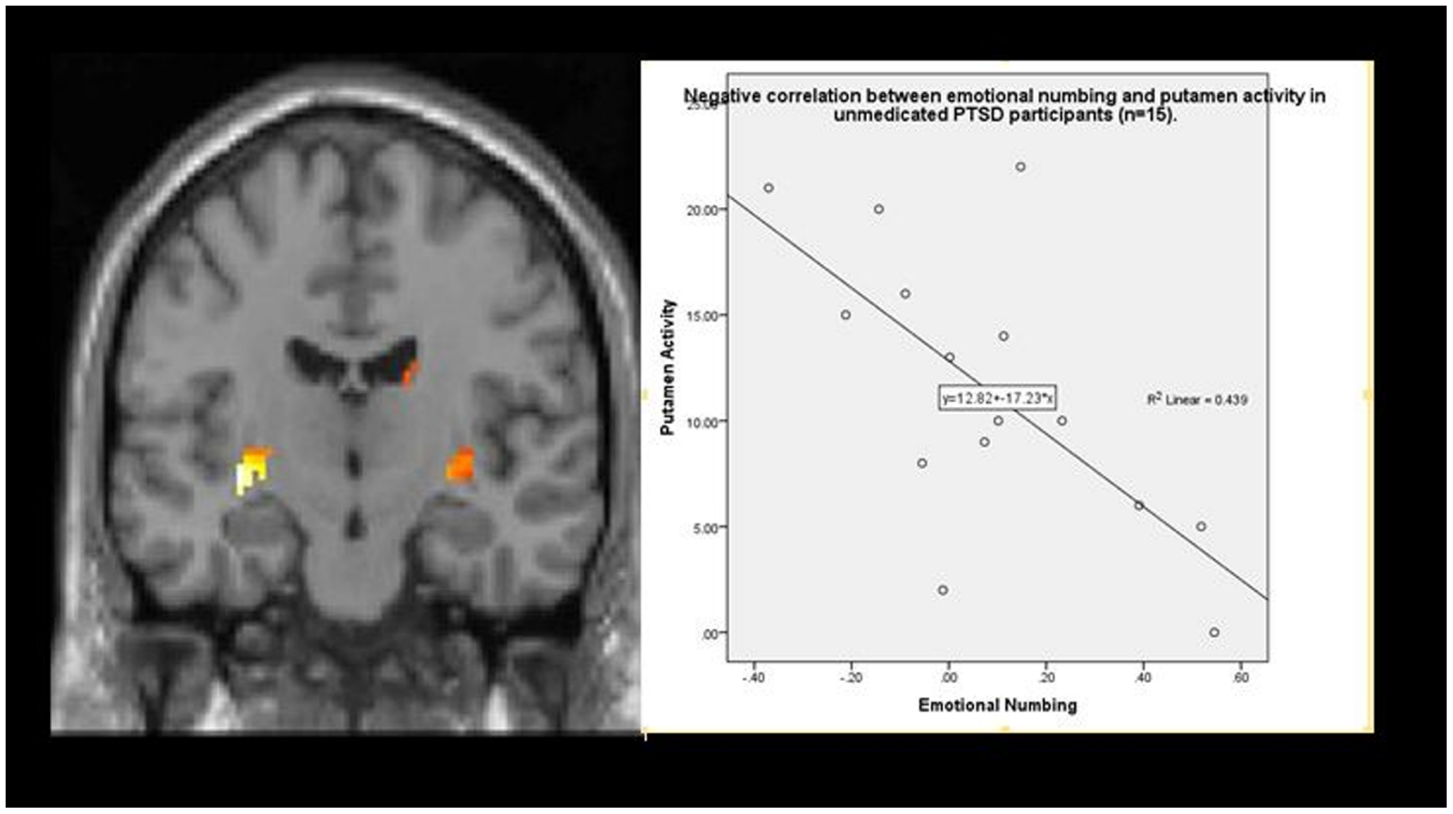
Correlations between BOLD activity to happy (-neutral) facial expressions in ventral striatum (putamen extending into nucleus accumbens) and CAPS emotional numbing scores in the unmedicated PTSD (n = 15) group.

Given the small samples in the depression sub-analysis, a correlation analysis was used to examine the relationships between emotional numbing symptoms and BOLD activation to happy faces in unmedicated PTSD participants, whilst taking depression and anxiety (DASS scales) and PTSD severity as covariates. Figure 3 presents a summary of findings. More severe symptoms of emotional numbing correlated negatively with activation in the right ventral striatum (putamen extending into nucleus accumbens: −32 −14 −4, 138 voxels, T = 5.7, p<.05, *r* = −.709) see Figure 3) and the culmen (−6 −52 −8, 219 voxels, T = 6.7,p<.01). A partial correlation analysis was also conducted between depression scores and activation in the right ventral striatum (putamen extending into nucleus accumbens (MNI coordinates) −32 −14 −4), whilst controlling for numbing scores. There was no significant correlation found between depression and ventral striatum activation (r = .27, p = .35).

## Discussion

Our hypotheses that activation in ventral striatal reward processing networks would be lower in PTSD to happy faces, and that this would be associated with emotional numbing symptoms, were largely confirmed. The PTSD group displayed behavioural evidence of less reactivity to happy faces as they rated happy faces as less intense in expression than trauma controls. Lower activation in ventral striatal regions (putamen and nucleus accumbens) and amygdala was seen in PTSD compared to Trauma Controls in response to happy (-neutral) faces. Importantly, a correlational analysis that controlled for depression, anxiety and PTSD severity found a negative correlation between emotional numbing symptoms in PTSD and activation in ventral striatum in the PTSD group, but there was no correlation between depression symptoms and ventral striatal activation.

These findings accord with recent reports of lower activation in nucleus accumbens and ventral striatal regions in PTSD in response to gains in monetary incentive tasks [9], [10], and extend this previous research by demonstrating that PTSD is associated with impaired perception of innate reward signals (happy facial expressions), with a concurrent reduction in activity within ventral striatal networks. This is significant given evidence that facial perception contributes significantly to social functioning [20], and that emotional numbing in PTSD is the most significant predictor of chronic functional and social impairment [5], [6]. Importantly, this study also extends previous research by including a trauma-exposed control group – our results suggest that lower activation in ventral striatal reward networks is specifically associated with PTSD, rather than trauma exposure per se.

Despite this convergence, there was also evidence of distinctive functional networks mediating motivational incentive tasks and happy face perception. Sailer and colleagues (2009) reported lower activation in medial frontal gyrus and nucleus accumbens in PTSD, reflecting weaker mesocortical dopaminergic activity [10]. Elman and colleagues (2009) found lower activation in both ventral and dorsal striatal regions (encompassing putamen and caudate, but *not* nucleus accumbens) [9]. In contrast, we found less activation *specifically* in ventral striatum (left nucleus accumbens extending into putamen) to happy faces. Ventral striatum is thought to process salience and prediction of reward signals, whereas dorsal striatum is more involved in action planning, cognitive function and sensorimotor integration [30], [31]. Nucleus accumbens is a region that is particularly associated with processing positive signals [32].

The fact that lower activation in ventral striatal activity did not remain significant when removing PTSD participants with depression from the analysis, suggests that depression may contribute to the impairments in these networks. This accord with recent evidence of reduced nucleus accumbens activation to monetary rewards in major depression [11]. However, depression is not likely to be a sufficient explanation for these null findings, as although the PTSD group with depression displayed a significant reduction in activation of ventral striatum, this reduction was at trend level in the PTSD without depression group. Further, direct comparison of the PTSD with and without depression groups revealed that the PTSD with depression group displayed lower activation in more dorsal regions ofthe striatum relative to the PTSD group without depression. This lower activation may reflect impaired activation in networks governing action initiation associated with depression [30]. The failure to find lower ventral striatal activation within the PTSD group without depression compared to controls is more likely a result of limited statistical power, as the sample size for non-depressed participants was small (n = 8). This interpretation is supported by the fact that the significant negative correlation between emotional numbing and ventral striatum remained significant after controlling for depression, and there was no significant correlation between ventral striatal activity and depression whilst controlling for numbing. Medication status does not appear to be a sufficient explanation for these findings, as comparisons between the Trauma Control group and medicated PTSD patients and unmedicated PTSD patients resulted in reduction of ventral striatal activation being reduced to trend level in both analyses. Again, however, this may relate to the smaller group sizes in these analyses. To clarify the role of depression and medication status, future research needs to examine depressed and non-depressed, medicated and non-medicated PTSD participants with larger group sizes.

There was a trend for reduced amygdala activation in the PTSD group compared to trauma controls which became significant once removing medicated PTSD participants, suggesting that medication use may have masked some amygdala effects. This reduction in amygdala activity to happy faces contrasts with reports of heightened amygdala activation to fearful faces in PTSD [33], [34], suggesting that these results may not be explained by a generalized impairment in processing emotional expressions. Amygdala activation has been associated with fear perception [35], but also with a broader construct of stimulus salience [36]. There is increasing evidence that the amygdala is also responsive to happy facial expressions [16]–[18], [37]. To the extent that amygdala activation reflects detection of salient stimuli [36], the fMRI findings suggest there is a blunted response to salient positive signals, in contrast to a heightened detection of threat (fearful face) stimuli in PTSD.

An unexpected but convergent finding was the evidence for lower activation in right orbitofrontal cortex in the non-depressed PTSD group compared to trauma controls in response to happy faces. Medial OFC has afferents to ventral striatum [38] and is thought to modulate reward related behaviours [39]–[43]. Anterolateral orbitofrontal cortex increases activity in response to signals of absence of reward (Ursu & Carter, 2005), and in anticipation of viewing aversive images [44], [45]. Therefore, this finding suggests that PTSD without comorbid depression is associated with lower activation in reward-processing networks in the orbitofrontal cortex.

To explore predictions of the prevailing model of emotional numbing [2], future research needs to examine responses to positive and threatening stimuli concurrently. The use of a passive viewing task with a predictable series of facial expressions may have reduced our BOLD signal, as fMRI activity has been found to be greatest in response to unpredictable rewarding stimuli in nucleus accumbens and medial orbitofrontal cortex [46]. Indeed, these findings should be viewed as preliminary and require replication, as they did not survive correction for multiple comparisons. Future studies examining responses to positive signals in depressed and non-depressed PTSD samples should employ larger sample sizes and a more active emotional task to enhance BOLD signal. A more comprehensive measure of emotional numbing should be employed in future studies rather than relying on PTSD symptom scores from the CAPS. Future research should examine group differences in BOLD responses to neutral facial expressions, independently of emotional faces. Although findings may be confounded by the direct comparison of happy and neutral faces [47], examining the percentage signal change for our key ventral striatal findings suggests the main effects are driven by response to the happy face rather than the neutral face (Figure 2). Future research should employ a separate fixation baseline condition to avoid a direct comparison of happy and neutral faces. Finally, the cross-sectional design prevented us identifying if altered brain patterns preceded or followed traumatic exposure.

In conclusion, this study provides novel evidence that individuals with PTSD perceive happy facial expressions as less intense and that lower activation in ventral striatal-limbic reward networks is associated with symptoms of emotional numbing in PTSD. Importantly, these blunted responses to positive facial signals are not accounted for by trauma exposure or comorbid depression. The identification of impaired functioning of reward networks in PTSD points to the need for better understanding of these networks in PTSD and the role they play in the trajectory of PTSD. Improving the capacity to respond to positive emotional signals in PTSD is of vital therapeutic importance, given evidence that emotional numbing symptoms contribute predominantly to functional and social impairments in chronic PTSD.
